# Defibrillators for prevention from sudden cardiac death: is it that easy?

**DOI:** 10.1093/europace/euaa044

**Published:** 2020-04-01

**Authors:** David Duncker, Christian Veltmann

**Affiliations:** Rhythmology and Electrophysiology, Department of Cardiology and Angiology, Hannover Medical School, Carl-Neuberg-Str. 1, 30625, Hannover, Germany

We have read with interest the 2019 EHRA consensus document on cardiac arrhythmias in the emergency settings of acute coronary syndrome and revascularization.^1^ Authors have to be congratulated to address this underrepresented topic and to give recommendations dealing with limited data in this special field. However, we would like to state that ‘limited data’ nevertheless deserves a differentiated interpretation of the anyhow available data.

Table 6 of the consensus document provides ‘optimal timing for implantable cardioverter-defibrillator (ICD) implantation after acute myocardial infarction (MI)’.^1^ Authors chose to classify optimal timing in three groups (<48 h, 48 h–40 days, and >40 days) in this setting. However, no study ever studied primary preventive ICD implantation in patients differentiating these categories. Accordingly, authors do not provide any reference for their recommendations. Two randomized studies evaluated effect of early implantation (<40 days) of ICD for primary prevention (DINAMIT and IRIS) and did not show a mortality benefit for early implantation. Therefore, since long, early ICD implantation is not recommended after acute MI.^2^ But furthermore, to allow reverse remodelling and decide about primary preventive ICD implantation, re-evaluation of left ventricular ejection fraction (LVEF) after 6–12 weeks was given a Class I recommendation in current ESC SCD guidelines.^2^ In a study from Sjöblom *et al*.^3^, there was a significant further improvement of LVEF between 1 and 3 months after acute MI suggesting that waiting for at least 3 months (not 40 days) is reasonable to avoid unnecessary ICD implantation. Importantly, a prerequisite for primary preventive ICD implantation in heart failure with reduced ejection fraction is stable optimized heart failure medication for at least 3 months.^2^^,^^4^ This circumstance is not achievable within 40 days post-MI. Unfortunately, this key element is not even mentioned in these recommendations.^1^

The authors also provide recommendations for the use of the ‘wearable’ defibrillator (WCD). Table 6 recommends that the WCD is not indicated in any of the described time frames post-MI.^1^

These consensus recommendation contradicts current ESC guidelines.^2^ Even when considering the results of the VEST study,^5^ these results do not allow any deductions on differentiated recommendations concerning timing since VEST included patients post-MI without considering any timing differentiation of 48 h, 40 days, or beyond 40 days. Regrettably, authors only use the primary endpoint of VEST^5^ to underpin the consensus recommendation ‘WCD not indicated’. In VEST, however, WCD wear time—being a key condition of this therapy—was unprecedentedly poor. Additionally, the trial showed further substantial limitations, including trial conduction, site selection, power calculation, remote monitoring/intervention, or endpoint adjudication. Thus, VEST does not represent an appropriate basis for this recommendation focusing only on the primary endpoint.

Post-MI patients should be carefully put on optimal medical therapy and re-evaluated after at least 3 months.^2^^,^^4^ In selected patients, a temporary protection seems to be reasonable in individual cases.^6^ Even if considering the only randomized trial on WCD to date,^5^ the available data should not lead to not recommend WCD use at all. The potential benefit may be life-saving for selected patients. Therefore, we should claim for new high-quality studies on patient selection, heart failure management, and WCD use in patients post-MI.

**Conflict of interest:** D.D. received lecture honorary, travel grants and/or a fellowship grant from Abbott, Astra Zeneca, Biotronik, Boehringer Ingelheim, Boston Scientific, Medtronic, Microport, Pfizer, and Zoll. C.V. received lecture honorary, travel grants and/or a fellowship grant from Abbott, Astra Zeneca, Bayer, Biotronik, Boehringer Ingelheim, Boston Scientific, Medtronic, Pfizer, and Zoll.
